# A small molecule targeting ALK1 prevents Notch cooperativity and inhibits functional angiogenesis

**DOI:** 10.1007/s10456-014-9457-y

**Published:** 2015-01-04

**Authors:** Georgina Kerr, Helen Sheldon, Apirat Chaikuad, Ivan Alfano, Frank von Delft, Alex N. Bullock, Adrian L. Harris

**Affiliations:** 1 Structural Genomics Consortium, University of Oxford, Old Road Campus Research Building, Roosevelt Drive, Headington, Oxford, OX3 7DQ UK; 2 Weatherall Institute of Molecular Medicine, John Radcliffe Hospital, University of Oxford, Headington, Oxford, OX3 9DS UK

**Keywords:** ALK1, ACVRL1, BMP9, Notch, Angiogenesis, Hypersprouting

## Abstract

Activin receptor-like kinase 1 (ALK1, encoded by the gene *ACVRL1*) is a type I BMP/TGF-β receptor that mediates signalling in endothelial cells via phosphorylation of SMAD1/5/8. During angiogenesis, sprouting endothelial cells specialise into tip cells and stalk cells. ALK1 synergises with Notch in stalk cells to induce expression of the Notch targets HEY1 and HEY2 and thereby represses tip cell formation and angiogenic sprouting. The ALK1-Fc soluble protein fusion has entered clinic trials as a therapeutic strategy to sequester the high-affinity extracellular ligand BMP9. Here, we determined the crystal structure of the ALK1 intracellular kinase domain and explored the effects of a small molecule kinase inhibitor K02288 on angiogenesis. K02288 inhibited BMP9-induced phosphorylation of SMAD1/5/8 in human umbilical vein endothelial cells to reduce both the SMAD and the Notch-dependent transcriptional responses. In endothelial sprouting assays, K02288 treatment induced a hypersprouting phenotype reminiscent of Notch inhibition. Furthermore, K02288 caused dysfunctional vessel formation in a chick chorioallantoic membrane assay of angiogenesis. Such activity may be advantageous for small molecule inhibitors currently in preclinical development for specific BMP gain of function conditions, including diffuse intrinsic pontine glioma and fibrodysplasia ossificans progressiva, as well as more generally for other applications in tumour biology.

## Introduction

The Notch and ALK1 signalling pathways play critical roles in the vasculature as evidenced by their respective linkage to the diseases Alagille syndrome [1] and hereditary haemorrhagic telangiectasia (HHT) [2]. Angiogenic stimuli such as VEGF induce quiescent endothelial cells to specialise into tip cells that migrate towards the stimulus and into attached stalk cells that proliferate behind for lumen formation. Dll4 expression on tip cells allows for the binding and activation of Notch receptors on adjacent stalk cells triggering the release of the Notch intracellular domain (NICD) for transcriptional activation. This signalling is critically required for lateral feedback inhibition, which blocks adjacent tip cell formation and therefore limits the number of extending filopodia [3, 4]. Recent work has highlighted a co-dependence on the type I BMP/TGF-β receptor kinase ALK1 [5, 6]. ALK1 signals via phosphorylation of the transcription factors SMAD1/5/8, which act synergistically with NICD to induce the expression of Notch target genes, including *HEY1* and *HEY2*.

Overexpression of Dll4 reduces neo-angiogenesis, but also differentiates vessels and downregulates VEGFR2 causing tumour resistance to anti-VEGF therapies [7]. Conversely, anti-Dll4 antibodies or gamma-secretase inhibitors that block Notch activation induce non-productive hypersprouting and reduce tumour growth [8, 9]. Targeting of ALK1 signalling has also generated significant interest as an anti-angiogenic therapy. Clinical trials are investigating anti-ALK1 antibodies, as well as soluble ALK1-Fc protein, which acts to sequester the receptor’s high-affinity ligand BMP9 [10].

In parallel, small molecule inhibitors of BMP signalling are being developed against the closely related kinase ALK2. Activating mutations in the intracellular domain of ALK2 are associated with the musculoskeletal disorder fibrodysplasia ossificans progressiva (FOP) [11] as well as the childhood cancer diffuse intrinsic pontine glioma (DIPG) [12–15]. We recently reported a 2-aminopyridine compound, K02288, that exhibited low nanomolar affinity for both ALK1 and ALK2, whilst retaining significant selectivity against the wider human kinome [16]. In contrast to dorsomorphin and LDN-193189, the inhibitor K02288 does not bind to the receptor kinase VEGFR2 and therefore forms a valuable tool to explore the effects of small molecule BMP inhibition in the endothelium. Here, we show that K02288 can inhibit BMP9-ALK1 signalling in human umbilical vein endothelial cells (HUVECs) and block the induction of both BMP9 and Notch-dependent target genes. As a result, K02288 induced a hypersprouting phenotype in 3D culture and caused dysfunctional vessel formation in an advanced CAM model of angiogenesis. Targeting ALK1 in angiogenesis may be synergistic with ALK2-targeted therapies which aim to address tumour growth or ectopic bone formation.

## Materials and methods

### Antibodies, recombinant proteins and chemicals

BMP9 was obtained from PeproTech and used at a final concentration of 1 or 10 ng/mL. Recombinant Dll4 was obtained from R&D Systems. Antibodies against SMAD1 (#9743), P-SMAD1/5/8 (#9511) and P-SMAD2 (#3101) were obtained from cell signalling. LDN-193189 was a kind gift from Dr Paul Yu (Harvard). K02288 was purchased from Biofocus and used at 1 μM. Soluble ALK1-Fc was purchased from R&D Systems and used at 100 ng/mL. The gamma-secretase inhibitor dibenzazepine (DBZ) was obtained from Calbiochem and used at 10 nM. The gamma-secretase inhibitor DAPT (*N*-[(3,5-difluorophenyl)acetyl]-l-alanyl-2-phenyl]glycine-1,1-dimethylethyl ester) was obtained from Tocris and used at a final concentration of 10 μM.

### Protein expression

Human ALK1 (UniProt P37023; residues 195–503) was cloned into the vector pFB-LIC-Bse. Baculoviral expression was performed in Sf9 insect cells. Cell pellets were resuspended in 50 mM HEPES pH 7.5, 500 mM NaCl, 5 mM imidazole and 5 % glycerol, supplemented with protease inhibitor set V (Calbiochem). Cells were lysed using a C5 high-pressure homogeniser (Emulsiflex). Nucleic acids were removed from the soluble lysate using DEAE-cellulose resin. ALK1 protein was purified using an N-terminal hexahistidine tag and eluted from nickel-Sepharose by a step-wise gradient of increasing imidazole up to 250 mM in a buffer comprising 50 mM HEPES pH 7.5, 500 mM NaCl, 5 % glycerol and 0.5 mM Tris-(2-carboxyethyl)phosphine (TCEP). The eluted protein was cleaved with TEV protease and further purified by size exclusion chromatography using a S200 HiLoad 16/60 Superdex column equilibrated in 50 mM Tris–HCl pH 7.5, 300 mM NaCl and 0.5 mM TCEP. The protein was then concentrated to 10 mg/mL with an additional 2 % glycerol.

### Structure determination

ALK1 protein was mixed with 1 mM LDN-193189 and crystallised using the sitting drop vapour diffusion method. Viable crystals were grown at 20 °C mixing 200 nL protein solution with 100 nL of a reservoir solution containing 16 % PEG3350, 0.2 M Na/KPO_4_, 5 % ethylene glycol and 2 % glycerol. On mounting, crystals were cryo-protected with an additional 25 % ethylene glycol. Diffraction data collected at Diamond Light Source beamline I24 were processed with XDS [17] and scaled with SCALA [18]. The coordinates of ALK2 (PDB 3H9R) were used in molecular replacement performed in Phaser [19]. The structure was subjected to manual model building in COOT [20] alternated with structure refinement in REFMAC [21], and the final model was verified for its geometric correctness with MolProbity [22]. Diffraction data and refinement statistics are provided in Table 1.

### Cell culture

HUVECs were obtained from Lonza, maintained in EGM2 (Lonza) and used for experiments between passage 3 and passage 6. For sDll4-coated plates, six-well plates were coated in 0.2 % gelatin (w/v) in PBS containing 1 μg/mL sDll4 or BSA control and incubated at 4 °C for 24 h before use. Plates were warmed to 37 °C and the coating solution aspirated prior to seeding HUVECs. HUVECs were treated for 30 min with chemical inhibitor or vehicle control before addition of BMP9. Cells were collected after 60 min for Western blot analysis or 4 h for qPCR analysis.

Where indicated, HUVECs were also grown on uncoated plates. These cells were treated with BMP9 alone or with BMP9 that was pre-incubated for 30 min with ALK1-Fc. Additionally, on some plates, HUVECs were starved for 6 h in low serum medium (EGM2 without foetal bovine serum). Complete medium was then added in the presence or absence of indicated inhibitors. Cells were harvested 45 min after treatment and analysed by Western blot.

### Transfections and dual luciferase assays

HUVECs were transfected with 800 ng RBPJ luciferase construct and 200 ng Renilla luciferase in a 10-cm dish using lipofectamine LTX reagent (Life Technologies). After 24 h, cells were replated in low serum medium in 24-well plates coated with sDll4 or BSA. Cells were allowed to attach for 5 h, K02288 added for 30 min and 10 ng/mL BMP9 added for a further 16 h. Dual luciferase assays were performed according to the manufacturer’s protocol (Promega).

### Western blotting

Cells were harvested and lysed in 20 mM Tris–HCl pH 7.5, 150 mM NaCl, 1 % Triton X-100, 25 mM NaF and protease inhibitors (Roche) on ice for 30 min. Protein concentration was determined by BCA assay (Pierce) and 15 µg run on 4–12 % Bis–Tris gel (Life Technologies). The protein was transferred onto PVDF membrane (GE Healthcare) and probed with the relevant antibody at 4 °C overnight. Protein bands were detected using ECL (Pierce) and an LAS4000 image reader.

**Table 1:** Data collection and refinement statistics

Complex	ALK1-LDN193189
PDB accession code	3MY0
*Data Collection*	
Beamline	Diamond light source, I24
Wavelength (Å)	0.9779
Resolution^a^ (Å)	58.96–2.65 (2.79–2.65)
Spacegroup	*P* 3_2_
Cell dimensions	*a* = *b* = 118.8, *c* = 510.8 Å
	*α* = *β* = 90.0°, *γ* = 120.0°
No. unique reflections^a^	233,884 (34,027)
Completeness^a^ (%)	99.9 (99.5)
*I*/*σI* ^a^	7.8 (2.0)
*R*_merge_^a^	0.153 (0.593)
Redundancy^a^	4.3 (3.0)
*Refinement*	
Ligands	LDN-193189
No. atoms in refinement (P/L/O)^b^	53,061/744/126
*R*_fact_ (%)	20.7
*R*_free_ (%)	24.7
*B*_factor_ (P/L/O)^b^ (Å^2^)	46/36/29
RMS deviation bond (Å)	0.012
RMS deviation angle (°)	1.3
*MolProbity*	
Ramachandran favour	94.7
Ramachandran allowed	99.2

*RMS* root mean square

^a^Values in brackets show the statistics for the highest-resolution shells

^b^P/L/O indicate protein, ligand molecules presented in the active sites and other (water and solvent molecules), respectively

### RNA prep and quantitative PCR

Total RNA was prepared using Trizol (Invitrogen) following the manufacturer’s instructions. Two micrograms of total RNA was converted to cDNA using Superscript III reverse transcriptase (Invitrogen). Triplicate wells were subjected to comparative quantitative PCR using SensiMix SYBR Low-ROX (Bioline) and gene-specific primers. Expression levels were normalised to GAPDH and relative dRn to UT sample calculated. Experiments were repeated at least three times, and error bars represent SEM. Primer sequences for qPCR are provided in Table 2.

**Table 2:** Sequences of primers used for qPCR (5′–3′)

ID1for	CTACGACATGAACGGCTGTTACTC
ID1rev	CTTGCTCACCTTGCGGTTCT
SMAD6for	TGAATTCTCAGACGCCAGCATGTC
SMAD6rev	ATGCCGAAGCCGATCTTGCTGC
HEY1for	CGAAATCCCAAACTCCGATA
HEY1rev	TGGATCACCTGAAAATGCTG
HEY2for	ATGAGCATAGGATTCCGAGAGTG
HEY2rev	GGCAGGAGGCACTTCTGAAG
JAG1for	ACTGTCAGGTTGAACGGTGTC
JAG1rev	ATCGTGCTGCCTTTCAGTTT
VEGFR1for	TCCCTTCCTTCAGTCATGTGT
VEGFR1rev	AAGAAGGAAACAGAATCTGCAA
VEGFR2for	CGGCTCTTTCGCTTACTGTT
VEGFR2rev	CCTGTATGGAGGAGGAGGAA

### Sprouting assays

HUVEC were grown as spheroids (500 cells/spheroid) and embedded in a fibrin gel as described by Nakatsu et al. [23]. K02288 or ALK1-Fc was added on top of the gel in EGM-2 media. The media was changed every 2 days. Quantification of sprout number and length was made 2 days after addition of inhibitor and images acquired after a further 4 days of treatment. Experiments were repeated at least three times, and error bars represent SEM.

### CAM assays

Fertilised chicken eggs (Henry Stewart & Co. Ltd) were incubated at 37 °C with a relative air humidity of 65 %. On embryo development day 3 (EDD 3), a hole of approximately 3 mm in diameter was opened in the eggshell, and on EDD 6, the hole in the shell was extended to a diameter of approximately 3 cm. A polyethylene ring was deposited on the CAM and 100 μL of either K02288, ALK1-Fc or PBS was pipetted inside the ring. After 4 more days (EDD 10), the vessels were visualised under a microscope and representative pictures acquired.

All experiments adhered to human and animal rights.

## Results

### Structural basis for small molecule inhibition of ALK1

**Fig. 1:**
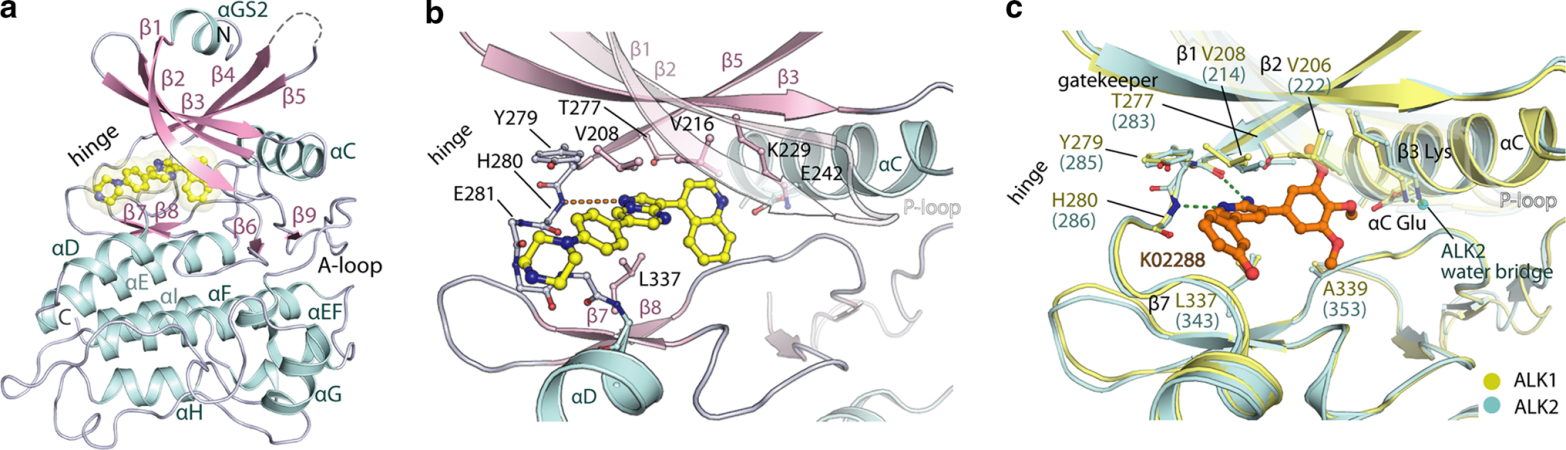
Structural basis for inhibition of the ALK1 kinase domain. **a** Ribbon representation of the ALK1 kinase domain highlighting the different secondary structural elements. The co-crystallised inhibitor LDN-193189 is bound to the hinge region in the ATP pocket. **b** Side chain interactions involved in the binding of LDN-193189. **c** Model for K02288 binding to ALK1 and ALK2. Shown is a superposition of ALK1 and the ALK2 co-crystal structure with K02288 (PDB 3MTF) [16]. A water molecule is shown for ALK2, whereas waters could not be built in the ALK1 structure due to the lower resolution of this structure

To date, structural investigations of the BMP receptors and their small molecule inhibitor binding have focussed on ALK2 [16, 24, 25]. To characterise the homologous structure of human ALK1, we expressed various deletion constructs in Sf9 insect cells and purified the resulting proteins for crystallisation trials. Viable crystals were obtained in the presence of LDN-193189 using a construct comprising ALK1 residues 195–503. The resulting structure was refined at 2.65 Å resolution and defines the kinase domain as well as seven residues from the N-terminal GS domain (Fig. 1a).

Overall, ALK1 adopts an inactive conformation of the kinase domain that is closely conserved with ALK2 (root-mean-square deviation 0.73 Å across 292 Cα atoms). The co-crystallised inhibitor LDN-193189 is bound as expected to the kinase hinge region with a single hydrogen bond to His280 (Fig. 1b). Residues lining the inhibitor binding pocket are strictly conserved between ALK1 and ALK2 explaining their common binding to small molecule BMP inhibitors, including K02288 (Fig. 1c) [16]. By similarity to the ALK2 co-structure (PDB 3MTF), K02288 is expected to bind ALK1 in an ATP-mimetic fashion with two hydrogen bonds to the kinase hinge (Fig. 1c). The trimethoxyphenyl specificity group occupies the central hydrophobic region and may hydrogen bond directly with the catalytic β3 lysine, or via a water molecule as observed in ALK2 (Fig. 1c) [16].

### K02288 inhibits the BMP9-ALK1 pathway

**Fig. 2:**
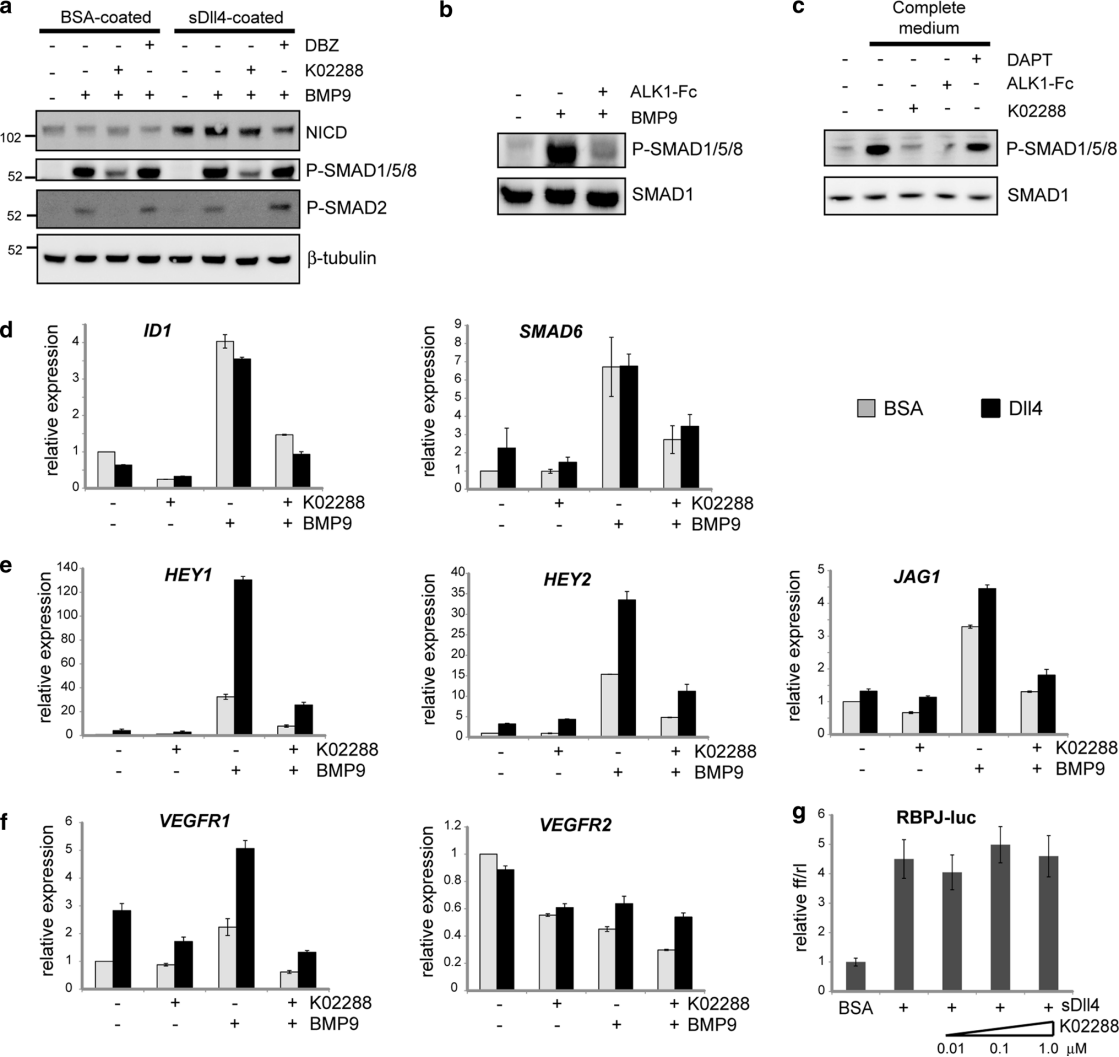
K02288 inhibits BMP9-ALK1 signalling in HUVECs. **a** HUVECs were seeded onto BSA- or Dll4-coated plates and treated with the indicated inhibitors and 10 ng/mL BMP9 for 1 h before collecting and analysing by Western blot. **b** HUVECs on uncoated plates were treated as indicated with 1 ng/mL BMP9 in the presence or absence of 100 ng/mL ALK1-Fc and similarly analysed by Western blot. **c** HUVECs on uncoated plates were starved in low serum medium (EGM2 without serum) for 6 h after which the medium was replaced with complete EGM2 in the presence or absence of the indicated inhibitors. Samples were analysed by Western blot. **d**–**f** Expression levels of BMP-responsive (**d**), Notch-responsive (**e**) and tip cell-specific (**f**) genes were determined in HUVECs seeded onto BSA- or Dll4-coated plates, 4 h after treatment with 1 μM K02288 and 1 ng/mL BMP9 (**d**) or 10 ng/mL BMP9 (**e**, **f**). **g** HUVECs were transiently transfected with a RBPJ-responsive luciferase construct for 24 h, re-plated onto BSA- or Dll4-coated plates in the presence or absence of K02288 and luciferase activity determined after 24 h

To assess the ability of K02288 to inhibit BMP9-ALK1 signalling in HUVECs, we analysed the downstream phosphorylation of SMAD1/5/8 by Western blot following treatment with inhibitors or vehicle control. Addition of K02288 reduced BMP9-induced P-SMAD1/5/8 levels, whereas the gamma-secretase inhibitor dibenzazepine (DBZ) had no effect (Fig. 2a). Stimulating HUVECs with Dll4 to activate the Notch pathway resulted in an induction in the levels of NICD that was reduced in the presence of DBZ (Fig. 2a). However, Dll4 stimulation had no effect on P-SMAD1/5/8 levels and did not interfere with the ability of BMP9 to activate SMAD1/5/8 or with the ability of K02288 to inhibit BMP9-induced P-SMAD1/5/8, suggesting that K02288 is specifically inhibiting BMP9-ALK1 signalling (Fig. 2a). In HUVECs, BMP9 can also phosphorylate SMAD2 through heteromeric complexes of ALK1/ActRII [26]. Again, K02288 was able to inhibit BMP9-induced P-SMAD2 independently of the Notch pathway (Fig. 2a). The established ligand trap ALK1-Fc was also able to inhibit P-SMAD1/5/8 formation stimulated by either BMP9 (Fig. 2b) or serum-rich complete medium (Fig. 2c). The latter was similarly inhibited by K02288, but not by the gamma-secretase inhibitor DAPT (Fig. 2c).

We also analysed the effect of K02288 on BMP9-induced gene expression using quantitative real-time PCR (Fig. 2d–f). As expected, upregulation of the BMP-response genes *ID1* and *SMAD6* was inhibited by K02288 similarly to the reduction observed in P-SMAD1/5/8 (Fig. 2d, grey bars). Additional stimulation of HUVECs with Dll4 had minimal effect (Fig. 2d, black bars). In contrast, maximal induction of the Notch-responsive genes *HEY1*, *HEY2* and *JAG1* required combined stimulation with both BMP9 and Dll4, consistent with the involvement of both the P-SMAD1/5/8 and the NICD pathways (Fig. 2e). K02288 treatment was effective at downregulating all three genes under all conditions (Fig. 2e). The expression of *VEGFR1*, a marker of tip cell specification, was similarly upregulated by BMP9 and Dll4, whereas *VEGFR2* was unchanged or slightly reduced (Fig. 2f). Again, K02288 treatment markedly reduced the ability of both stimuli to induce *VEGFR1* (Fig. 2f). Finally, to exclude the possibility that K02288 inhibits Dll4-Notch transcriptional activity via the co-activator RBPJ, we tested the effect of K02288 in a RBPJ-responsive dual luciferase reporter assay (Fig. 2g). As expected, there was no effect of K02288 over a 100-fold concentration range. Together, these data suggest that K02288 inhibits BMP and Notch target gene expression by inhibiting the BMP receptor ALK1 as well as ALK2.

### K02288 induces a hypersprouting phenotype in HUVECs

**Fig. 3:**
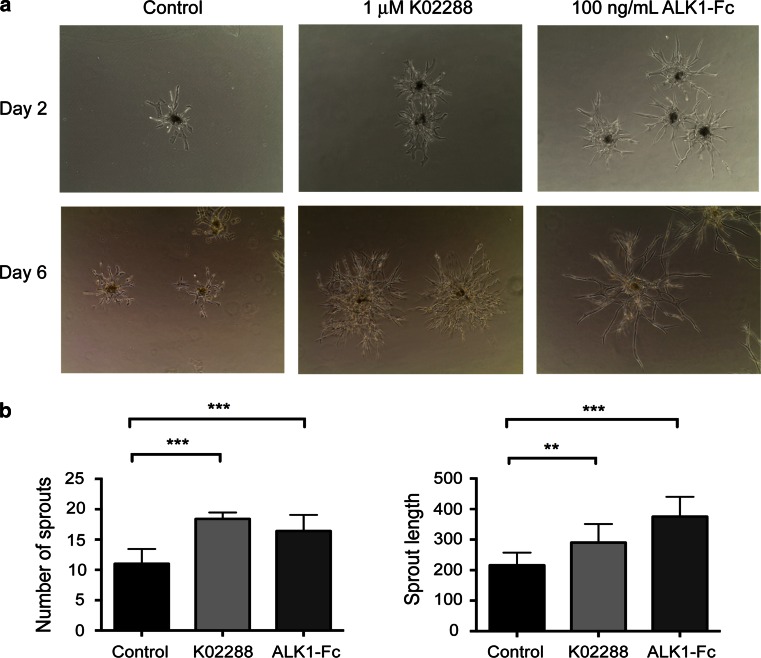
K02288 treatment results in a hypersprouting phenotype. **a** Endothelial spheroids were embedded in a fibrin gel and treated with either 1 μM K02288 or 100 ng/mL ALK1-Fc. The vehicle DMSO was used as a control. Ten spheroids per condition were photographed at ×10 magnification on day 2 or day 6 after embedding (representative examples are shown). **b** The number and length of sprouts were quantified on day 2 after embedding using ImageJ software. Experiments were carried out at least three times, and *error bars* represent SEM. Data were analysed using a one-way ANOVA; **p < 0.01, ***p < 0.001

We next analysed the effects of K02288 in 3D culture models of angiogenesis. We used a hanging drop assay in which endothelial cells are collected as spheroids and embedded in a fibrin gel. In this assay, treatment with K02288 resulted in a hypersprouting phenotype (Fig. 3a), with an increase in both the number and the length of vessels formed after 2 days (Fig. 3b). Hypersprouting was also observed upon treatment with ALK1-Fc, consistent with the work of Larrivée et al. [5] and in agreement with the expected cooperation between BMP9 and Notch signalling [5, 6]. Interestingly, Cunha et al. [27] have also reported an inhibitory effect of ALK1-Fc, perhaps reflecting that sprouting is both a highly dynamic and context dependent process.

### K02288 causes dysfunctional angiogenesis in a chick embryo CAM model

**Fig. 4:**
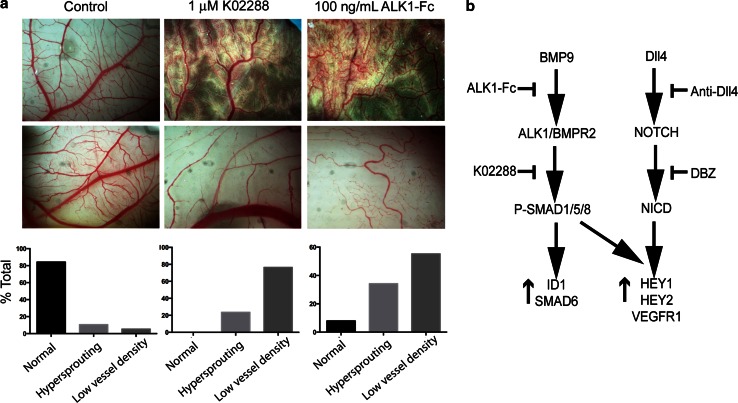
K02288 induces dysfunctional angiogenesis in a chick embryo CAM model. **a** Twenty eggs were treated per condition, and representative images acquired at EDD 10. The images were sorted blind into three categories; normal, hypersprouting and low vessel density and the percentage in each category calculated. **b** Schematic overview of BMP9 and Dll4 signalling showing the intervention points of different pathway inhibitors

The hypersprouting effects induced by K02288 were reminiscent of those observed upon disruption of the Notch pathway [8], suggesting that K02288 increased tip cell specification potentially resulting in dysfunctional vessel formation. To assess whether K02288 may interfere with angiogenesis in vivo, we used chick embryo chorioallantoic membrane (CAM) models which allow easy visualisation and quantification of angiogenesis. Again, the effects of K02288 were similar to those observed with ALK1-Fc (Fig. 4a). Two distinct phenotypes of disrupted angiogenesis were observed with both treatments. A subset of the treated CAM models displayed hypersprouting consistent with the 3D culture models. Moreover, shadows and halos were observed around the angiogenic sprouts suggestive of leaky dysfunctional vessels. A large fraction of the CAM models exhibited a distinctive phenotype of low vessel density reflecting the dysfunctional angiogenesis which occurs following hypersprouting (Fig. 4a). Thus, the small molecule inhibitor K02288 has potential to inhibit angiogenesis in vivo similarly to ALK1-Fc.

## Discussion

Here, we show that a small molecule inhibitor targeting intracellular BMP receptor domains can interfere with angiogenesis in a similar fashion to ALK1-Fc and Notch pathway inhibitors consistent with the known functional synergy between these two signalling pathways (Fig. 4b). Current pre-clinical development of small molecule BMP inhibitors is directed primarily at ALK2 for treatment of the skeletal malformation disorder FOP and more recently the brain tumour DIPG. Both diseases are associated with recurrent activating mutations in the ALK2 intracellular domain that render the gain of function resistant to endogenous biological inhibitors, including noggin. The ATP pockets of the ALK1 and ALK2 kinase domains are strictly conserved creating a significant challenge for the design of chemical inhibitors that selectively target either protein. Indeed, in drosophila, ALK1 and ALK2 are represented by a single ortholog, saxophone. However, angiogenesis is a potentially critical process for the ectopic bone formation characteristic of FOP, as well as the tumour growth in DIPG. Therefore, the combined inhibition of both receptors may have incremental benefits.

Whilst Notch inhibition has shown promising anti-tumour activity, it is also associated with potential toxicity [28], raising some concerns for the development of BMP inhibitors that may similarly impact this pathway. BMP signalling is also complex and pleiotropic with wide ranging roles outside the vasculature in both development and tissue repair. Nonetheless, such molecules appear to be well tolerated in animals [25]. The availability of multiple tool compounds and therapeutic strategies offers promise to increase our understanding of vascular biology and the hope to develop safe and effective new medicines.
